# The Global Prevalence of *Strongyloides stercoralis* Infection

**DOI:** 10.3390/pathogens9060468

**Published:** 2020-06-13

**Authors:** Dora Buonfrate, Donal Bisanzio, Giovanni Giorli, Peter Odermatt, Thomas Fürst, Christina Greenaway, Michael French, Richard Reithinger, Federico Gobbi, Antonio Montresor, Zeno Bisoffi

**Affiliations:** 1 Department of Infectious, Tropical Diseases and Microbiology, IRCCS Sacro Cuore Don Calabria Hospital, Negrar, 37024 Verona, Italy; federico.gobbi@sacrocuore.it (F.G.); zeno.bisoffi@sacrocuore.it (Z.B.); 2 RTI International, Washington, DC 20005, USA; dbisanzio@rti.org (D.B.); mfrench@rti.org (M.F.); reithinger@rti.org (R.R.); 3 Epidemiology and Public Health Division, School of Medicine, University of Nottingham, Nottingham NG7 2UH, UK; 4 Centre for Experimental Medicine & Rheumatology, William Harvey Research Institute, Barts & The London School of Medicine & Dentistry, Queen Mary University of London, London E1 4NS, UK; giovanni.giorli@gmail.com; 5 Swiss Tropical and Public Health Institute, CH-4051 Basel, Switzerland; peter.odermatt@swisstph.ch (P.O.); thomas.fuerst@swisstph.ch (T.F.); 6 University of Basel, CH-4051 Basel, Switzerland; 7 Division of Infectious Diseases and Clinical Epidemiology, Sir Mortimer B. Davis-Jewish General Hospital, McGill University, Montreal, QC H3A 2B2, Canada; ca.greenaway@mcgill.ca; 8 Department of Control of Neglected Tropical Diseases, World Health Organization, 1211 Geneva, Switzerland; montresora@who.int; 9 Department of Diagnostics and Public Health, University of Verona, 37129 Verona, Italy

**Keywords:** strongyloides, strongyloidiasis, prevalence, epidemiology

## Abstract

Strongyloidiasis is a common neglected tropical disease in tropical and sub-tropical climatic zones. At the worldwide level, there is high uncertainty about the strongyloidiasis burden. This uncertainty represents an important knowledge gap since it affects the planning of interventions to reduce the burden of strongyloidiasis in endemic countries. This study aimed to estimate the global strongyloidiasis prevalence. A literature review was performed to obtain prevalence data from endemic countries at a worldwide level from 1990 to 2016. For each study, the true population prevalence was calculated by accounting for the specificity and the sensitivity of testing and age of tested individuals. Prediction of strongyloidiasis prevalence for each country was performed using a spatiotemporal statistical modeling approach. The country prevalence obtained from the model was used to estimate the number of infected people per country. We estimate the global prevalence of strongyloidiasis in 2017 to be 8.1% (95% CI: 4.2–12.4%), corresponding to 613.9 (95% CI: 313.1–910.1) million people infected. The South-East Asia, African, and Western Pacific Regions accounted for 76.1% of the global infections. Our results could be used to identify those countries in which strongyloidiasis prevalence is highest and where mass drug administration (MDA) should be deployed for its prevention and control.

## 1. Introduction

Strongyloidiasis is the infection caused by the soil-transmitted helminth (STH) *Strongyloides stercoralis*. Its global prevalence was previously estimated at 30–100 million infected people [1], but these estimates were subsequently questioned in light of diagnostic issues that characterized the few studies available at that time [2]. Moreover, neither the source nor the methods which formed the basis for these estimates were reported in the paper. Indeed, the diagnostic methods commonly used in the field for other STHs, such as Kato–Katz and direct smear examination, have a very low sensitivity for *S. stercoralis* [3]. The Baermann method and Koga agar plate culture (APC) have a higher sensitivity than stool microcopy, but still miss a large proportion of infections [3]; polymerase chain reaction (PCR) is highly specific, but not more sensitive than the Baermann method and APC [4]. Serology is the most sensitive method, although false positive results are possible, due to cross-reactions and long-term persistence of antibodies [3]. Overall, none of the available diagnostic tests can be considered the gold standard for the diagnosis of strongyloidiasis. Recently, a paper estimated the prevalence of strongyloidiasis as a ratio to hookworm, in order to partly overcome the diagnostic issue [5].

The morbidity caused by *S. stercoralis* is not well defined compared to other STHs. A systematic review evaluated the clinical burden caused by strongyloidiasis and reported that urticaria (reported by 33% of infected individuals in the included studies), abdominal pain (62%), and diarrhea (50%) might be frequently affecting people with strongyloidiasis [6]. Although the results of the review were limited by the paucity of studies focusing on this topic, the clinical relevance of *S. stercoralis* infection cannot be disregarded, because in immunosuppressed individuals it can lead to a syndrome (hyperinfection/dissemination) that is invariably fatal if not promptly and properly cured and is often fatal despite treatment [7]. Ivermectin (IVM) is the drug of choice for the treatment of *S. stercoralis* infection [7], and it has been recently included in the WHO list of essential medicines for this purpose [8]. Unfortunately, IVM is often not easily available outside specific donation programs aimed at the elimination of lymphatic filariasis (LF) and onchocerciasis.

Currently, no specific strategies for the control of *S. stercoralis* infection have been implemented in endemic areas. This is mostly due to the knowledge gap regarding the global prevalence of the disease and the difficult access to quality-assured and affordable IVM.

In this work, we aimed to estimate the prevalence of strongyloidiasis at a global and country level, using a spatiotemporal statistical modeling approach.

## 2. Results

### 2.1. Review of the Literature

**Figure 1:**
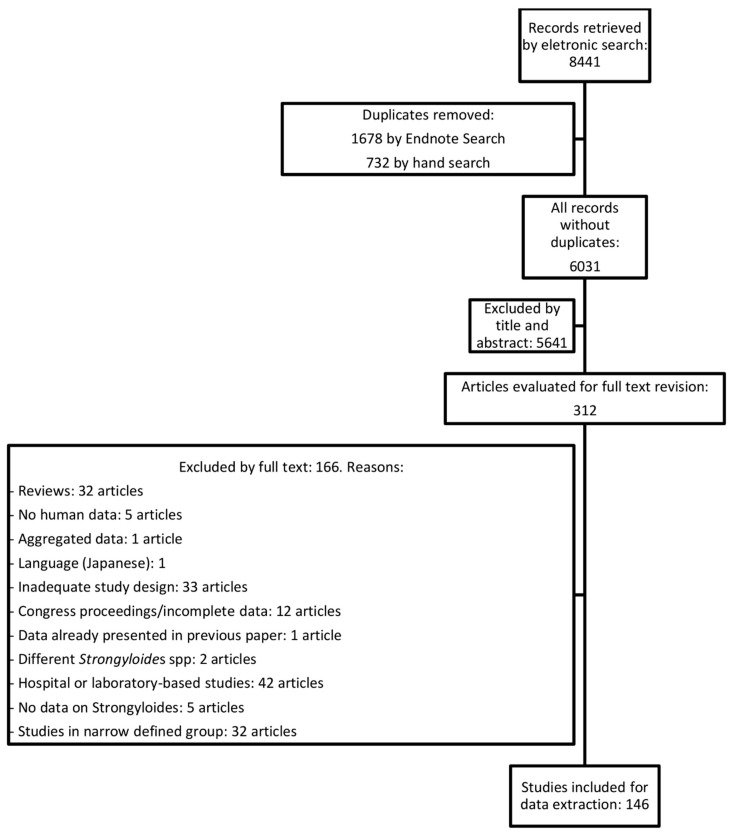
Flow chart describing the review process.

**Figure 2:**
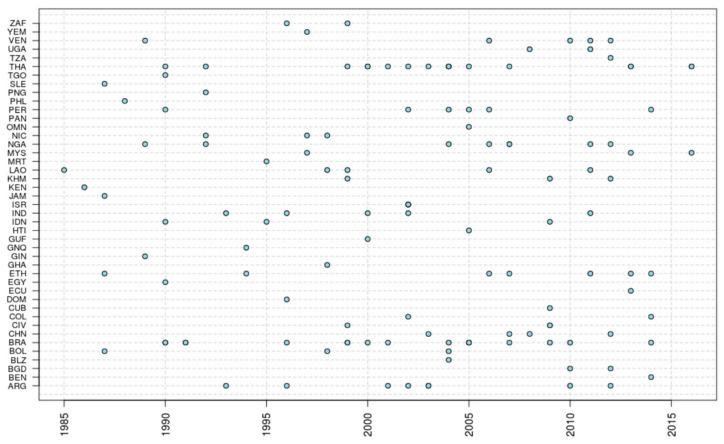
Year of survey reported in the research publications included in the literature review by country. In the Y-axis, country codes are reported according to ISO 3 (legend available in Table S2).

The flow of the literature review is described in Figure 1. The review of the literature identified 146 articles (Supplementary File S1) with data on the prevalence of strongyloidiasis from 43 countries (Figure 2). Brazil and Thailand were the countries with the highest number of studies. Twenty studies used either the Baermann method or stool culture (including agar plate, Harada Mori or any other cultural method) as diagnostic methods; a combination of diagnostic tests including Baermann method and/or stool culture was used in 22 studies. PCR was used in 7 studies, and a further 2 studies used it in combination with other tests. Serology was used in 16 studies, and a further 3 studies used serology in combination with other tests. The remaining studies used less sensitive diagnostic methods (mostly Kato–Katz and single/multiple direct smears). The prevalence reported was adjusted considering the diagnostic test used, as described above in the Methods section.

### 2.2. Global and Regional (WHO) Prevalence According to the Model

The best model describing strongyloidiasis prevalence included GDP, percentage of rural population, territory roughness, sanitation, annual mean temperature, and annual precipitation:

STG-PR = GDP + RURAL + RUG + SANIT + TEMP + RAIN + REGION_RND_ (1)

This model was used to estimate strongyloidiasis prevalence for each country at the worldwide level with a 95% confidence interval (95% CI).

**Figure 3:**
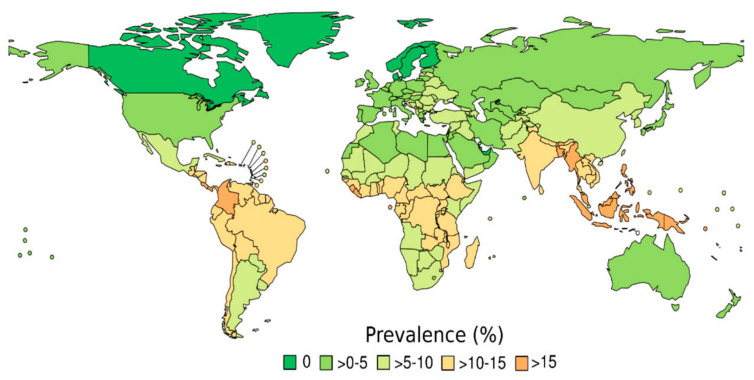
Estimated strongyloidiasis prevalence (STG-PR) for 2017, as predicted by the best statistical model.

Strongyloidiasis prevalence and number of infected people are reported at the world and regional levels in Table 1 and Figure 3. 

In 2017, the estimated strongyloidiasis prevalence was 8.1% (95% CI: 4.2%–12.4%), which corresponds to 613.9 (95% CI: 313.1–910.1) million people infected with Strongyloides. Referring to the WHO regions, the highest number of infected people live in the South-East Asia Region (SEAR) estimated at 237.3 (95% CI: 129.9–353.3) million, followed by Western Pacific Region (WPR) with 133.2 (95% CI: 68.1–198.4) million, and African Region (AFR) with 108.1 (95% CI: 55.1–160.9) million; combined this represents 76.1% of the total infected population worldwide (Table 1).

At the regional level, high *S. stercoralis* prevalence was estimated for countries in AFR (10.3%; 95% CI: 5.3–15.3%), for the Americas Region (AMR) (6.9%; 95% CI: 3.5–10.2%), and SEAR (12.1%; 95% C.I.: 6.1–17.9%). In the AMR, countries in Central America and the northern part of South America had the highest prevalence (Figure 3), particularly Panama (15.7%; 95% CI: 8–23.4%), Costa Rica (15.7%; 95% CI: 8–23.4%), and Colombia (18.4%; 95% CI: 9.4–27.4%). S. stercoralis prevalence in AFR was estimated to be high in sub-Saharan countries, with prevalence in West Africa higher compared with the rest of the AFR countries and Sierra Leone (17%; 95% CI: 8.7–25.3%), Liberia (16%; 95% CI: 8.4–24.6%), and Sao Tome and Principe (20.7%; 95% CI: 10.6–30.8%) having particularly high estimated prevalence. All countries in the SEAR had high levels of strongyloidiasis prevalence, with the highest prevalence estimated for Myanmar (19.2%; 95% CI: 9.8–28.6%). Low prevalence of infection (<0.1%) was estimated for high-income countries in temperate zones, with countries at the northern latitudes having the lowest prevalence. Prevalence estimates at country level are reported in Table S1.

## 3. Discussion

Different to the previous review on the global prevalence [9], here we provide new estimates of *S. stercoralis* prevalence at a global level. Our results suggest that, similar to other STHs, a large number of people are infected by *S. stercoralis* worldwide and they are mostly distributed in sub-Saharan Africa, Latin America and East Asia [10]. The global prevalence resulting from our modeling is ten times higher than previous estimates, ranging between 30 to 100 million people [1]. Although these estimates are regularly cited by articles on *S. stercoralis*, we were unable to find any evidence base for these estimates as well as for previous (assessing the prevalence at 3 to 30 million cases [1,11]) (under)estimation. This may be the main reason why *S. stercoralis* infection has lagged behind other STHs in being addressed in countries’ STH control programming. Recently, a systematic review and meta-analysis [12] on schistosomiasis and *S. stercoralis* prevalence in migrants from endemic countries to non-/low-endemic countries (defined as the United States, Canada, Australia, New Zealand, Western Europe, and Israel) reported similar prevalence figures to ours for the migrants’ main geographic areas of origin. 

*S. stercoralis* infection affects an important proportion of the world population and this calls for action. Not surprisingly, almost all cases of the severe, usually fatal form of the disease are reported, precisely, in non-endemic countries, with very few cases reported from the highly-endemic geographic areas [13]. This means that most of the deaths caused by this parasite are simply undetected. Moreover, besides the disseminated disease that is caused by immune suppression in chronically infected patients, the clinical burden of the chronic, uncomplicated *S. stercoralis* infection is still poorly known [6,14], reflecting the paucity of studies and the general lack of funding for research on this parasite.

The number of studies reporting data on the prevalence of *S. stercoralis* infection is still scarce and sparse in time and space. This paucity of data with very few country-level surveys is the main limitation in our study. Furthermore, many studies included in our review still relied on diagnostic tests with a low sensitivity which would have resulted in an underestimation of the prevalence. Adjustment for test accuracy and a robust model that could fill the gap in data in many areas were necessary. Because we did not have enough data to have time series of testing results from the same location for a long period, we were not able to account in our model for the effect of MDA based on IVM targeting other parasites.

A consensus should be reached on priority research areas that could support operational *S. stercoralis* prevention and control programming. If no action is taken a preventable disease will keep on taking thousands if not hundreds of thousands of lives. Indeed, because *S. stercoralis* causes a long-lasting infection, the proportion of infections in adults is higher than in children. While IVM is effective against *S. stercoralis* infection, a mass drug administration program only for *S. stercoralis* would be difficult to implement due to cost constraints. Ad hoc cost–benefit analyses might help to identify the best strategies to tackle *S. stercoralis* operationally, either on its own or in combination/integrated with other worm infections.

At least in a preliminary phase, prior to operational activities being implemented based on prevalence data from studies conducted one or two decades ago, it would be important to conduct surveys to estimate the prevalence of *S. stercoralis* in specific (e.g., high burden) areas and confirm modeling outputs presented here. There is an urgent need for guidelines indicating the optimal diagnostic methods for such surveys to allow for homogeneous and reliable estimates of prevalence.

## 4. Materials and Methods 

Prevalence data was modeled based on data retrieved from a literature review and a number of sources that provide data on key predictors of *S. stercoralis* prevalence. The review of the literature was performed in May 2017 in three databases (PubMed, WHOLIS, ISI Web of Science), using the MeSH terms “Strongyloides” and “Strongyloidiasis”, with no date of publication or language restrictions. The methods for the literature search are described in Supplementary File S2.

Country population data were obtained from the World Bank website [15], including total population, percentage of the population living in rural areas, and the fraction of the population by age, up to age 14 and older than 14 years. Additional data on the gross domestic product (GDP) per capita and percentage of GDP spent on health (for all sectors and the public sector) were collected from the World Bank website. Strongyloidiasis prevalence is also linked with the level of sanitation in a country [16]. To include this information in our analyses, we accessed the percentage of the population with access to a proper latrine from the UNICEF website [17].

Environmental factors also affect strongyloidiasis prevalence [18]. To account for environmental characteristics of each country, we included in our analyses the terrain ruggedness index [19] and land use characteristics (i.e., the percentage of the total country area that is desert, agricultural, and forest). These data were collected from the FAO website and Nunn and Puga [20]. *S. stercoralis* larvae living in soil have a high chance of surviving in humid and warm weather [21]. To account for country climate suitability for strongyloidiasis prevalence in our analyses, we included country data on annual average temperature and total annual rainfall from the World Bank website. 

All tabular data were imported into a database based on SQLite [22]. Geographical data were processed using QGIS [23], and statistical analyses performed using the R language [24] through the RStudio interface [25]. All the analyses were performed using free and open-source software installed on a Linux Mint 18 platform.

### 4.1. Estimation of Country Prevalence

To estimate the final country prevalence of strongyloidiasis, we had to adjust the data reported in each survey to the accuracy of the diagnostic test used and the age of tested individuals. The reported strongyloidiasis prevalence extracted through the literature review was adjusted using the specificity and sensitivity of each diagnostic test, reported by studies comparing the different diagnostic tests [25] and systematic reviews [3,4,26]. In particular, the following ranges of sensitivity were considered: direct stool examination/Kato–Katz: 5–21% [26]; formol ethyl-acetate concentration technique (FECT): 9–48% [4,26]; Baermann method and APC: 45–89% [26]; PCR: 62–72% [4]; IFAT: 81–98% [25]; ELISA crude antigen: 73–100 [25]; NIE-ELISA: 71–84% [25]; LIPS: 84–97% [25]. We performed the accuracy adjustment using the methods described in the scientific literature [27]. This correction used the direct relation between prevalence, sensitivity and specificity (details in Supplementary File S3).

**Figure 4:**
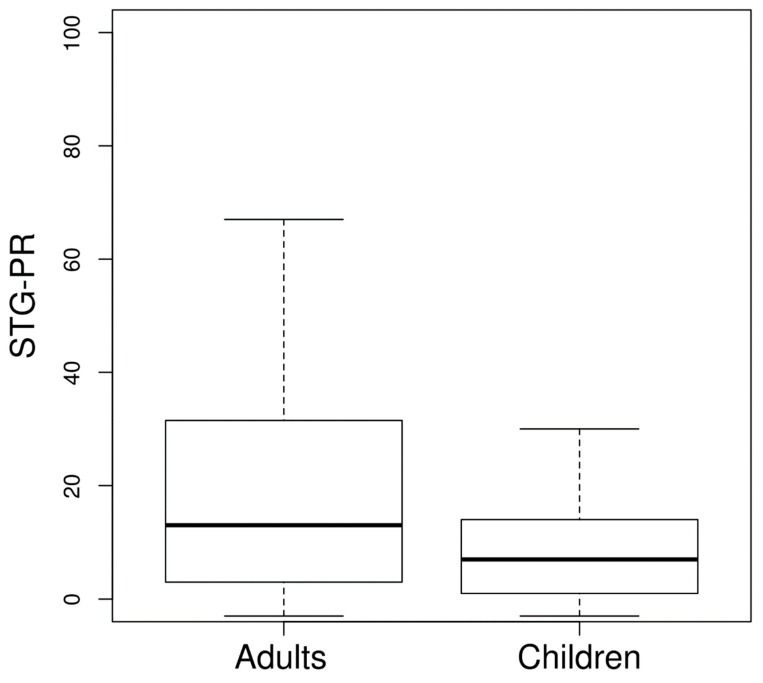
Boxplot of strongyloidiasis prevalence (STG-PR) in adults (≥15 years of age) and children. The prevalence showed in the graph was adjusted by test accuracy.

For each country, a final weighted mean of the adjusted prevalence by the sample size of each study was computed. After adjusting by test accuracy, we needed to calculate the all-age strongyloidiasis prevalence at the country level. Many studies did not sample all age groups but focused on particular ones. In order to estimate strongyloidiasis prevalence for the entire population of countries without an all-age prevalence, we separately estimated prevalence for children and adults. We considered two age groups: children < 15 years of age and individuals ≥ 15 years of age. We compared the infection prevalence of these two age groups (Figure 4) to compute the groups’ prevalence ratio. The ratio was obtained by calculating the mean of the ratio of the prevalence of the two age groups using those studies in which both age groups were sampled. The ratio was used as an adjusting factor to obtain the unknown strongyloidiasis prevalence of one of the age groups based on the known prevalence of the other age group. The prevalence of the two age groups was used to calculate the strongyloidiasis prevalence of the entire country’s population. The calculation took into consideration the proportion of the population belonging to the two age groups.

### 4.2. Statistical Methods

A model approach was implemented to calculate the strongyloidiasis prevalence at the worldwide level for the year 2017. A generalized linear mixed model (GLMM) was used to investigate the relationship of strongyloidiasis prevalence with economic and environmental factors [28]. The model was built using variables that could have an effect on strongyloidiasis prevalence levels in a country:

STG-PR = GDP + GDPHealth + EDU + RURAL + CROP + FOREST + RUG + SANIT + TEMP + RAIN + REGION_RND_ (2)

where GDP is per capita gross domestic product, GDPHealth is the percentage of GDP allocated to health expenditure, EDU is the percentage of the population who attended primary education, RURAL is the percentage of the population living in a rural setting, CROP is the percentage of the country’s land allocated for agriculture, FOREST is the percentage of the country’s land covered by forest, RUG is the ruggedness index of the country, SANIT is the percentage of the population with access to a proper latrine, TEMP is the mean annual temperature, RAIN is the total annual rainfall, and REGION_RND_ is the region as a random effect.

To determine the most important variable associated with strongyloidiasis prevalence, a model selection approach was applied to identify which variable had the highest ability to predict strongyloidiasis prevalence. A set of possible models were created, starting from the full model formula and varying the included variables (Table S2). Model selection was performed using the Akaike Information Criteria (AIC) of each model. The model with the lowest AIC was identified as the best one and was used to predict strongyloidiasis prevalence for the year 2017 [29].

## 5. Conclusions

Global prevalence of *S. stercoralis* is probably higher than previously thought. Ad hoc surveys should be carried out in areas where prevalence is estimated as high, prior to designing operational programs to control *S. stercoralis*.

## Figures and Tables

**Table 1:** Global and regional S. stercoralis prevalence (STG-PR), the number of infected individuals.

*WHO Region*	*STG-PR (95% CI)*	*Population Infected (95% CI) [Million]*
*AFR*	*10.3% (5.3–15.3)*	*108.1 (55.1–160.9)*
*AMR*	*6.9% (3.5–10.2)*	*69.8 (35.5–103.9)*
*EMR*	*5.8% (2.9–8.6)*	*39.4 (20.1–58.8)*
*EUR*	*2.8% (1.4–4.1)*	*26.1 (13.3–38.8)*
*SEAR*	*12.1% (6.1–17.9)*	*237.3 (129.9–353.3)*
*WPR*	*7.13% (3.6–10.6)*	*133.2 (68.1–198.4)*
*World*	*8.1% (4.2–12.4%)*	*613.9 (313.1–910.1)*

AFR: African Region; AMR: American Region; EMR: Eastern Mediterranean Region; EUR: European Region; SEAR: South-East Asia Region; WPR: Western Pacific Region.

## Supplementary Materials

The following are available online at https://www.mdpi.com/2076-0817/9/6/468/s1, File S1: References of papers included in the analysis, File S2: Review protocol, File S3: Prevalence adjustment based on diagnostic test. Table S1: Strongyloidiasis prevalence (STG-PR) and 95% CI for the countries for which estimates were made, Table S2: Summary of tested STAR models to assess the relationship of STG-PR with model variables.
