# Supplementary material for: The Global Prevalence of Strongyloides stercoralis Infection
Source: Pathogens. 2020 Jun 13;9(6):468. doi: 10.3390/pathogens9060468 (PMC7349647; doi:10.3390/pathogens9060468)
Supplement: Supplementary file 1 [file pathogens-09-00468-s001.zip › pathogens-812962-supplementary/pathogens-812962-suppl/Supplementary file S1.docx]

**References of papers included in the analysis**

1. [Abah](https://pubmed.ncbi.nlm.nih.gov/?sort=date&term=Abah+AE&cauthor_id=26600945),A.E., Arene F.O.I. (2015). “Status of Intestinal Parasitic Infections Among Primary School Children in Rivers State, Nigeria”. Journal of Parasitology Research. 2015: 937096. doi: 10.1155/2015/937096. Epub 2015 Oct 27.
2. Abdi, M., et al. (2017). "Prevalence of intestinal helminthic infections and malnutrition among schoolchildren of the Zegie Peninsula , northwestern Ethiopia." Journal of Infection and Public Health 10(1): 84-92.
3. Adeoye, G. O., et al. (2007). "Epidemiological studies of intestinal helminthes and malaria among children in Lagos, Nigeria." Pakistan journal of biological sciences : PJBS 10(13): 2208-2212.
4. Agbolade, O. M., et al. (2007). "Intestinal helminthiases and schistosomiasis among school children in an urban center and some rural communities in southwest Nigeria." 45(3): 233-238.
5. Aguiar Ji, A., et al. (2007). "Intestinal protozoa and helminths among Terena Indians in the State of Mato Grosso do Sul : high prevalence of Blastocystis hominis Protozoários e helmintos intestinais entre índios Terena do Estado do Mato Grosso do Sul : alta prevalência de Blastocystis." 40(6): 631-634.
6. Ahmad, A. F., et al. (2013). "Serological and molecular detection of Strongyloides stercoralis infection among an Orang Asli community in Malaysia." 2811-2816.
7. Aimpun, P. and P. Hshieh (2004). "Survey for intestinal parasites in Belize, Central America." The Southeast Asian journal of tropical medicine and public health 35(3): 506-511.
8. Akogun, O.B. (1989). “Some Social Aspects of Helminthiasis Among the People of Gumau District, Bauchi State, Nigeria”. J Trop Med Hyg 92(3):193-6.
9. Al-Hindi, A. I. (2002). Prevalence of intestinal parasites among school children in Deir El-Balah Town in Gaza Strip, Palestine. 22: 273-275.
10. Amor, A., et al. (2016). "High prevalence of Strongyloides stercoralis in school-aged children in a rural highland of north-western Ethiopia: the role of intensive diagnostic work-up." Parasites & vectors 9(1): 617-617.
11. Anantaphruti, M. T., et al. (2000). "Strongyloides stercoralis infection and chronological changes of other soil-transmitted helminthiases in an endemic area of southern Thailand." The Southeast Asian journal of tropical medicine and public health 31(2): 378-382.
12. Anantaphruti, M. T., et al. (2004). "Soil-transmitted helminthiases and health behaviors among schoolchildren and community members in a west-central border area of Thailand." The Southeast Asian journal of tropical medicine and public health 35(2): 260-266.
13. A Bracho, et al. (2014). [Parasitosis intestinales en niños y adolescentes de la etnia Yukpa de Toromo, estado Zulia, Venezuela. Comparación de los años 2002 y 2012](javascript:void(0)). Kasmera 42 (1), 41-51
14. Anosike, J. C., et al. (2004). "Prevalence of parasitic diseases among nomadic Fulanis of south-eastern Nigeria." Annals of agricultural and environmental medicine : AAEM 11(2): 221-225.
15. Anselmi, M., et al. (2015). "Mass Administration of Ivermectin for the Elimination of Onchocerciasis Significantly Reduced and Maintained Low the Prevalence of Strongyloides stercoralis in Esmeraldas, Ecuador." PLoS neglected tropical diseases 9(11): e0004150-e0004150.
16. Aplogan, A., et al. (1990). "[Digestive parasitic diseases in young children in a tropical outpatient setting]." Annales de pediatrie 37(10): 677-681.
17. Appleton, C. C. and E. Gouws (1996). "The distribution of common intestinal nematodes along an altitudinal transect in KwaZulu-Natal, South Africa." Annals of tropical medicine and parasitology 90(2): 181-188.
18. Appleton, C. C., et al. (1999). "The distribution of helminth infections along the coastal plain of Kwazulu-Natal province, South Africa." Annals of tropical medicine and parasitology 93(8): 859-868.
19. Arakaki, T., et al. (1992). "Age-related prevalence of Strongyloides stercoralis infection in Okinawa, Japan." Tropical and geographical medicine 44(4): 299-303.
20. Arakaki, T., et al. (1992). "Epidemiological aspects of Strongyloides stercoralis infection in Okinawa, Japan." The Journal of tropical medicine and hygiene 95(3): 210-213.
21. Arakaki, T., et al. (1992). “Is the prevalence of HTLV-1 infection higher in Strongyloides carriers than in non-carriers?” Trop Med Parasitol 43(3):199-200.
22. Ashford, R. W. and E. A. Atkinson (1992). "Epidemiology of Blastocystis hominis infection in Papua New Guinea: age-prevalence and associations with other parasites." Annals of tropical medicine and parasitology 86(2): 129-136.
23. Ashford, R. W., et al. (1992). "Polyparasitism on the Kenya coast. 1. Prevalence, and association between parasitic infections." Annals of tropical medicine and parasitology 86(6): 671-679.
24. Assis, E. M. d., et al. (2013). "[Prevalence of intestinal parasites in the Maxakali indigenous community in Minas Gerais, Brazil, 2009]." Cadernos de saude publica 29(4): 681-690.
25. Auer, C. (1990). "Health status of children living in a squatter area of Manila, Philippines, with particular emphasis on intestinal parasitoses." The Southeast Asian journal of tropical medicine and public health 21(2): 289-300.
26. Bangs, M. J., et al. (1996). "Intestinal parasites of humans in a highland community of Irian Jaya, Indonesia." Annals of tropical medicine and parasitology 90(1): 49-53.
27. Bartoloni, A., et al. (1990). "[Iron deficiency in an area of Bolivia and high prevalence of intestinal helminthiasis]." Parassitologia 32(3): 335-338.
28. Basualdo, J. A., et al. (2007). "Intestinal parasitoses and environmental factors in a rural population of Argentina, 2002-2003." Revista do Instituto de Medicina Tropical de Sao Paulo 49(4): 251-255.
29. Becker, S. L., et al. (2015). "Real-time PCR for detection of Strongyloides stercoralis in human stool samples from Côte d'Ivoire: diagnostic accuracy, inter-laboratory comparison and patterns of hookworm co-infection." Acta tropica 150: 210-217.
30. Becker, S. L., et al. (2011). "Diagnosis, clinical features, and self-reported morbidity of Strongyloides stercoralis and hookworm infection in a Co-endemic setting." PLoS neglected tropical diseases 5(8): e1292-e1292.
31. Beltramino, D., et al. (2003). "[Selective vs. mass treatment with antihelminthic drugs: experience in two hyperendemic communities]." Revista panamericana de salud publica = Pan American journal of public health 13(1): 10-18.
32. Bencke A, A. G. L. d. R. R. S. B. N. L. (2007). "Enteroparasitosesem escolares residentes na periferia." Revista De Patologia Tropical / Journal of Tropical Pathology 35(1): 31-36.
33. Bencke, A., et al. (2006). "EM ESCOLARES RESIDENTES NA PERIFERIA." 35(51): 31-36.
34. Birrie, H., et al. (1994). "Intestinal helminthic infections in the southern Rift Valley of Ethiopia with special reference to schistosomiasis." East African medical journal 71(7): 447-452.
35. Boia, M. N., et al. (1999). "[Cross-sectional study of intestinal parasites and Chagas' disease in the Municipality of Novo Airão, State of Amazonas, Brazil]." Cadernos de saude publica 15(3): 497-504.
36. Boko, P. M., et al. (2016). "Schistosomiasis and Soil Transmitted Helminths Distribution in Benin: A Baseline Prevalence Survey in 30 Districts." PloS one 11(9): e0162798-e0162798.
37. Boonjaraspinyo, S., et al. (2013). "A cross-sectional study on intestinal parasitic infections in rural communities, northeast Thailand." The Korean journal of parasitology 51(6): 727-734.
38. Borda, C. E., et al. (1996). "Intestinal parasitism in San Cayetano, Corrientes, Argentina." Bulletin of the Pan American Health Organization 30(3): 227-233.
39. Bosman, A., et al. (1991). "Prevalence and intensity of infection with intestinal parasites in areas of the Futa Djalon, Republic of Guinea." Parassitologia 33(2-3): 203-208.
40. Brandelli, C. L. C., et al. (2012). "Intestinal parasitism and socio-environmental factors among Mbyá-Guarani Indians, Porto Alegre, Rio Grande do Sul, Brazil." Revista do Instituto de Medicina Tropical de Sao Paulo 54(3): 119-122.
41. Buonfrate D, B. M. A. F. (2016). "Epidemiology of Strongyloides stercoralis in northern Italy: results of a multicentre case–control study, February 2013 to July 2014." Euro Surveill. 21(21): 30310-30310.
42. Cabada, M. M., et al. (2014). "Prevalence of soil-transmitted helminths after mass albendazole administration in an indigenous community of the Manu jungle in Peru." Pathogens and global health 108(4): 200-205.
43. Cancrini, G., et al. (1998). "Seroprevalence of Toxocara canis-IgG antibodies in two rural Bolivian communities." Parassitologia 40(4): 473-475.
44. Carme, B., et al. (2002). "Intestinal parasitoses among Wayampi Indians from French Guiana." Parasite (Paris, France) 9(2): 167-174.
45. Carvalho, G. L. X. d., et al. (2012). "A comparative study of the TF-Test®, Kato-Katz, Hoffman-Pons-Janer, Willis and Baermann-Moraes coprologic methods for the detection of human parasitosis." Memorias do Instituto Oswaldo Cruz 107(1): 80-84.
46. Cavuoti, D. and K. R. Lancaster (1992). "Intestinal parasitism of children on Corn Island, Nicaragua." The Pediatric infectious disease journal 11(9): 775-776.
47. Chacin-Bonilla, L., et al. (1992). "Prevalence of Entamoeba histolytica and other intestinal parasites in a community from Maracaibo, Venezuela." Annals of tropical medicine and parasitology 86(4): 373-380.
48. Chacín Bonilla, L., et al. (1990). "The problem of intestinal parasitosis in Venezuela” Invest Clin. PMID: 2090262 Spanish.
49. Chai, J. Y. and B. Hongvanthong (1998). "A small-scale survey of intestinal helminthic infections among the residents near Pakse, Laos." The Korean journal of parasitology 36(1): 55-58.
50. Champetier de Ribes, G., et al. (2005). "[Intestinal helminthiasis in school children in Haiti in 2002]." Bulletin de la Societe de pathologie exotique (1990) 98(2): 127-132.
51. Charlesworth, J., et al. (2019). "Osteoarthritis- a systematic review of long-term safety implications for osteoarthritis of the knee." BMC musculoskeletal disorders 20(1): 151-151.
52. Chhakda, T., et al. (2006). "Intestinal parasites in school-aged children in villages bordering Tonle Sap Lake, Cambodia." The Southeast Asian journal of tropical medicine and public health 37(5): 859-864.
53. Choubisa, S. L., et al. (2012). "Intestinal parasitic infection in Bhil tribe of Rajasthan, India." Journal of parasitic diseases : official organ of the Indian Society for Parasitology 36(2): 143-148.
54. Cimino, R. O., et al. (2015). "Identification of human intestinal parasites affecting an asymptomatic peri-urban Argentinian population using multi-parallel quantitative real-time polymerase chain reaction." Parasites & vectors 8: 380-380.
55. Djohan, V., et al. (2010). “Epidemiology of anguillulosis among public school children in Abidjan, Cote d'Ivoire”. Med Trop (Mars) 70(3): 305-306.
56. dos Santos, J. I., et al (1996). “The low sensitivity of the larval culture method (Harada-Mori) in the diagnosis of strongyloidiasis”. Revista da Sociedade Brasileira de Medicina Tropical 29(1): 51-52.
57. Edouard, A. (2004). “Evolution in the prevalence of intestinal parasitosis in the Fort de France University Hospital (Martinique). Presse Medicale 33(11):707-709.
58. Fernandez, M. C., et al. (2002). "A comparative study of the intestinal parasites prevalent among children living in rural and urban settings in and around Chennai." The Journal of communicable diseases 34(1): 35-39.
59. Fontanet, A.L. et al. (2000). “Epidemiology of infections with intestinal parasites and human immunodeficiency virus (HIV) among sugar-estate residents in Ethiopia”. Annals of Tropical Medicine and Parasitology 94(3): 269-278.
60. Gbakima, A. A. (1994). “Intestinal helminth infections in rural school-children in Njala, Sierra-Leone”. East African Medical Journal 71(12): 792-796.
61. Giraldi, N. et al. (2001). “Enteroparasites prevalence among daycare and elementary school children of municipal schools, Rolandia, PR, Brazil”. Revista da Sociedade Brasileira de Medicina Tropical 34(4): 385-387.
62. Goncalves, J.F. (1990). “Parasitological and serological studies on amebiasis and other intestinal parasitic infections in the rural sector around Recife, Northeast Brazil”. Revista Do Instituto De Medicina Tropical De Sao Paulo 32(6): 428-435.
63. Hall, A. et al. (1994). “Strongyloides stercoralis in an urban slum community in Bangladesh: factors independently associated with infection”88(5):527-530.
64. Hasegawa, H. et al. (1992). “Intestinal parasitic infections in Likupang, North Sulawesi, Indonesia”. 23(2): 219-227.
65. Incani, R. N., et al. (2017). "Diagnosis of intestinal parasites in a rural community of Venezuela: Advantages and disadvantages of using microscopy or RT-PCR." Acta tropica 167: 64-70.
66. Khieu, V., et al. (2014). “Strongyloides stercoralis infection and re-infection in a cohort of children in Cambodia”. Parasitology International 63(5): 708-712.
67. Machado, E.R., et al. (2008). “Enteroparasites and commensals among children in four peripheral districts of Uberlandia, State of Minas Gerais”. Revista Da Sociedade Brasileira De Medicina Tropical 41(6):581-585.
68. Natividad Carpio, I. (2007). "Presencia de Strongyloides stercoralis en un estudio sobre enteroparasitosis en escolares del asentamiento humano "La Candelaria", distrito de Chancay, provincia de Huaral, departamento de Lima." Acta méd. peruana [online]. 24(3): 177-180.
69. Neres-Norberg, A., et al. (2014). "[Intestinal Parasitism in Terena Indigenous People of the Province of Mato Grosso do Sul, Brazil]." Revista de salud publica (Bogota, Colombia) 16(6): 859-870.
70. Ngui, R., et al. (2016). "Epidemiological Characteristics of Strongyloidiasis in Inhabitants of Indigenous Communities in Borneo Island, Malaysia." The Korean journal of parasitology 54(5): 673-678.
71. Niamnuy, N., et al. (2016). "PREVALENCE AND ASSOCIATED RISK FACTORS OF INTESTINAL PARASITES IN HUMANS AND DOMESTIC ANIMALS ACROSS BORDERS OF THAILAND AND LAO PDR: FOCUS ON HOOKWORM AND THREADWORM." The Southeast Asian journal of tropical medicine and public health 47(5): 901-911.
72. Nithikathkul, C., et al. (2003). "Parasitic infections among Karen in Kanchanaburi Province, western Thailand." The Southeast Asian journal of tropical medicine and public health 34 Suppl 2: 86-89.
73. Nontasut, P., et al. (2005). "Prevalence of strongyloides in Northern Thailand and treatment with ivermectin vs albendazole." The Southeast Asian journal of tropical medicine and public health 36(2): 442-444.
74. Nyantekyi, L. A., et al. (2011). "Intestinal parasitic infections among under-five children and maternal awareness about the infections in Shesha Kekele, Wondo Genet, Southern Ethiopia." Ethiopian Journal of Health Development 24.
75. Oberhelman, R. A., et al. (1998). "Correlations between intestinal parasitosis, physical growth, and psychomotor development among infants and children from rural Nicaragua." The American journal of tropical medicine and hygiene 58(4): 470-475.
76. Oliveira, M. C. l., et al. (2003). "Intestinal parasites and commensals among individuals from a landless camping in the rural area of Uberlândia, Minas Gerais, Brazil." Revista do Instituto de Medicina Tropical de Sao Paulo 45(3): 173-176.
77. Olivera Rivero Mj, R. A. M. C. L. M. (2014). "Detección de Strongyloides stercoralis en Tierralta, Colombia, utilizando cuatro métodos parasitológicos." Revista cubana de medicina tropical 66(2): 202-209.
78. Onwuliri, C. O., et al. (1992). "Human helminthosis in a rural community of Plateau State, Nigeria." Angewandte Parasitologie 33(4): 211-216.
79. Ordóñez, L. E. and E. S. Angulo (2004). "[Efficacy of ivermectin in the treatment of children parasitized by Strongyloides stercoralis]." Biomedica : revista del Instituto Nacional de Salud 24(1): 33-41.
80. Patel, P. K. and R. Khandekar (2006). "Intestinal parasitic infections among school children of the Dhahira Region of Oman." Saudi medical journal 27(5): 627-632.
81. Pereira, A. P. M. F., et al. (2012). "The influence of health education on the prevalence of intestinal parasites in a low-income community of Campos dos Goytacazes, Rio de Janeiro State, Brazil." Parasitology 139(6): 791-801.
82. Perez, E., et al. (2000). "[Intestinal parasite infections and schistosomiasis in a poor urban area, in townships of the sugar cane belt and in villages of the semi-arid area of North-East Brazil]." Sante (Montrouge, France) 10(2): 127-129.
83. Pezzani, B. C., et al. (1996). "[Intestinal parasite infections in a periurban community from the Province of Buenos Aires, Argentina]." Boletin chileno de parasitologia 51(1-2): 42-45.
84. Pitisuttithum, P., et al. (1990). "Socio-economic status and prevalence of intestinal parasitic infection in Thai adults residing in and around Bangkok metropolis." Journal of the Medical Association of Thailand = Chotmaihet thangphaet 73(9): 522-525.
85. Prociv, P. and R. Luke (1993). "Observations on strongyloidiasis in Queensland aboriginal communities." The Medical journal of Australia 158(3): 160-163.
86. Rahmah, N., et al. (1997). "Parasitic infections among aborigine children at Post Brooke, Kelantan, Malaysia." The Medical journal of Malaysia 52(4): 412-415.
87. Raja'a, Y. A., et al. (2001). "Some aspects in the control of schistosomosis and soil-transmitted helminthosis in Yemeni children." Saudi medical journal 22(5): 428-432.
88. Rivero de R, Z., et al. (2009). “Prevalence of Enteroparasites, Rotavirus and Adenovirus in Apparently Healthy Children”.*Kasmera* 37(1): 62-73. ISSN 0075-5222
89. Rocha, R. S., et al. (2000). "[Assessment of schistosomiasis and other intestinal parasitoses in school children of the Bambuí municipality, Minas Gerais, Brazil]." Revista da Sociedade Brasileira de Medicina Tropical 33(5): 431-436.
90. Roche, J. and A. Benito (1999). "Prevalence of intestinal parasite infections with special reference to Entamoeba histolytica on the island of Bioko (Equatorial Guinea)." The American journal of tropical medicine and hygiene 60(2): 257-262.
91. Rodríguez, J. and J. Calderón (1991). "[Intestinal parasitosis in pre-school children from Tarapoto]." Revista de gastroenterologia del Peru : organo oficial de la Sociedad de Gastroenterologia del Peru 11(3): 153-160.
92. Rojas, C. L., et al. (2012). "[Second national survey of intestinal parasitic infections in Cuba, 2009]." Revista cubana de medicina tropical 64(1): 15-21.
93. Roldán, W. H., et al. (2009). "Frequency of human toxocariasis in a rural population from Cajamarca, Peru determined by DOT-ELISA test." Revista do Instituto de Medicina Tropical de Sao Paulo 51(2): 67-71.
94. Rowlins, S.C., et al. (1991). “Parasitic Infections in Young Jamaicans in Different Ecological Zones of the Island”. Trop Geogr Med 43(1-2):136-41.
95. Ruankham, W., et al. (2014). "Prevalence of helminthic infections and risk factors in villagers of Nanglae Sub-District, Chiang Rai Province, Thailand." Journal of the Medical Association of Thailand = Chotmaihet thangphaet 97 Suppl 4: S29-35.
96. Salem, S. A., et al. (1990). "A survey for enteroparasites in Menoufia governorate, Egypt with special reference to Strongyloides stercoralis." Journal of the Egyptian Society of Parasitology 20(1): 335-344.
97. Salim, N., et al. (2014). "Enterobiasis and strongyloidiasis and associated co-infections and morbidity markers in infants, preschool- and school-aged children from rural coastal Tanzania: a cross-sectional study." BMC infectious diseases 14: 644-644.
98. Sandoval, N. R., et al. (2015). "A survey of intestinal parasites including associated risk factors in humans in Panama." Acta tropica 147: 54-63.
99. Sanprasert, V., et al. (2016). "PREVALENCE OF INTESTINAL PROTOZOAN INFECTIONS AMONG CHILDREN IN THAILAND: A LARGE-SCALE SCREENING AND COMPARATIVE STUDY OF THREE STANDARD DETECTION METHODS." The Southeast Asian journal of tropical medicine and public health 47(6): 1123-1133.
100. Sato, Y., et al. (1991). "Gelatin particle indirect agglutination test for mass examination for strongyloidiasis." Transactions of the Royal Society of Tropical Medicine and Hygiene 85(4): 515-518.
101. Sato, Y., et al. (1990). "Application of enzyme-linked immunosorbent assay for mass examination of strongyloidiasis in Okinawa, Japan." International journal for parasitology 20(8): 1025-1029.
102. Sawaya, A. L., et al. (1990). "The risk approach in preschool children suffering malnutrition and intestinal parasitic infection in the city of São Paulo, Brazil." Journal of tropical pediatrics 36(4): 184-188.
103. Sayasone, S., et al. (2015). "Repeated stool sampling and use of multiple techniques enhance the sensitivity of helminth diagnosis: a cross-sectional survey in southern Lao People's Democratic Republic." Acta tropica 141(Pt B): 315-321.
104. Schär, F., et al. (2014). "The prevalence and diversity of intestinal parasitic infections in humans and domestic animals in a rural Cambodian village." Parasitology international 63(4): 597-603.
105. Schär, F., et al. (2013). "Evaluation of real-time PCR for Strongyloides stercoralis and hookworm as diagnostic tool in asymptomatic schoolchildren in Cambodia." Acta tropica 126(2): 89-92.
106. Shield, J., et al. (2015). "Intestinal parasites of children and adults in a remote Aboriginal community of the Northern Territory, Australia, 1994-1996." Western Pacific surveillance and response journal : WPSAR 6(1): 44-51.
107. Singh, H. L., et al. (2004). "Helminthic infestation of the primary school-going children in Manipur." The Journal of communicable diseases 36(2): 111-116.
108. Singh, S., et al. (1993). "Trichuris vulpis infection in an Indian tribal population." The Journal of parasitology 79(3): 457-458.
109. Sithithaworn, J., et al. (2005). "Comparative assessment of the gelatin particle agglutination test and an enzyme-linked immunosorbent assay for diagnosis of strongyloidiasis." Journal of clinical microbiology 43(7): 3278-3282.
110. Sithithaworn, P., et al. (2003). "Epidemiology of Strongyloides stercoralis in north-east Thailand: application of the agar plate culture technique compared with the enzyme-linked immunosorbent assay." Transactions of the Royal Society of Tropical Medicine and Hygiene 97(4): 398-402.
111. Sithithaworn, P., et al. (2006). "Epidemiology of food-borne trematodes and other parasite infections in a fishing community on the Nam Ngum reservoir, Lao PDR." The Southeast Asian journal of tropical medicine and public health 37(6): 1083-1090.
112. Sousa-Figuereido, J.C., et al. (2012). “Epidemiology of Malaria, Schistosomiasis, Geohelminths, Anemia and Malnutrition in the Context of a Demographic Surveillance System in Northern Angola”. PloS One <https://doi.org/10.1371/journal.pone.0033189>.
113. Steinmann, P., et al. (2015). "Control of soil-transmitted helminthiasis in Yunnan province, People's Republic of China: experiences and lessons from a 5-year multi-intervention trial." Acta tropica 141(Pt B): 271-280.
114. Steinmann, P., et al. (2007). "Occurrence of Strongyloides stercoralis in Yunnan Province, China, and comparison of diagnostic methods." PLoS neglected tropical diseases 1(1): e75-e75.
115. Stothard, J. R., et al. (2008). "Strongyloides stercoralis: a field-based survey of mothers and their preschool children using ELISA, Baermann and Koga plate methods reveals low endemicity in western Uganda." Journal of helminthology 82(3): 263-269.
116. Štrkolcová, G., et al. (2017). "The roundworm Strongyloides stercoralis in children, dogs, and soil inside and outside a segregated settlement in Eastern Slovakia: frequent but hardly detectable parasite." Parasitology research 116(3): 891-900.
117. Sultana, Y., et al. (2012). "Seroepidemiology of Strongyloides stercoralis in Dhaka, Bangladesh." Parasitology 139(11): 1513-1520.
118. Sultana, Y., et al. (2012). "Strongyloidiasis in a high risk community of Dhaka, Bangladesh." Transactions of the Royal Society of Tropical Medicine and Hygiene 106(12): 756-762.
119. Tang, N. and N. J. Luo (2003). "A cross-sectional study of intestinal parasitic infections in a rural district of west China." The Canadian journal of infectious diseases = Journal canadien des maladies infectieuses 14(3): 159-162.
120. Tanner, S., et al. (2009). "Influence of helminth infections on childhood nutritional status in lowland Bolivia." American journal of human biology : the official journal of the Human Biology Council 21(5): 651-656.
121. Taranto, N. J., et al. (2003). "Clinical status and parasitic infection in a Wichí Aboriginal community in Salta, Argentina." Transactions of the Royal Society of Tropical Medicine and Hygiene 97(5): 554-558.
122. Teklehaymanot, T. (2006). "Intestinal parasitosis among Kara and Kwego semi- pastoralist tribes in lower Omo Valley , Southwestern Ethiopia." 0-5.
123. Toma, A., et al. (1999). "Questionnaire survey and prevalence of intestinal helminthic infections in Barru, Sulawesi, Indonesia." The Southeast Asian journal of tropical medicine and public health 30(1): 68-77.
124. Toma, H., et al. (2000). "Community control studies on Strongyloides infection in a model island of Okinawa, Japan." The Southeast Asian journal of tropical medicine and public health 31(2): 383-387.
125. Tungtrongchitr, A., et al. (2007). "The potential usefulness of the modified Kato thick smear technique in the detection of intestinal sarcocystosis during field surveys." The Southeast Asian journal of tropical medicine and public health 38(2): 232-238.
126. Téllez, A., et al. (1997). "Prevalence of intestinal parasites in the human population of León, Nicaragua." Acta tropica 66(3): 119-125.
127. Ugbomoiko, U. S. and I. E. Ofoezie (2007). "Multiple infection diagnosis of intestinal helminthiasis in the assessment of health and environmental effect of development projects in Nigeria." Journal of helminthology 81(3): 227-231.
128. Urbani, C., et al. (1997). "[Intestinal parasitic infections and schistosomiasis in the valley of the Senegal river in the Islamic Republic of Mauritania]." Medecine tropicale : revue du Corps de sante colonial 57(2): 157-160.
129. Vannachone, B., et al. (1998). "An epidemiological survey on intestinal parasite infection in Khammouane Province, Lao PDR, with special reference to Strongyloides infection." The Southeast Asian journal of tropical medicine and public health 29(4): 717-722.
130. Verhagen, L. M., et al. (2013). "High malnutrition rate in Venezuelan Yanomami compared to Warao Amerindians and Creoles: significant associations with intestinal parasites and anemia." PloS one 8(10): e77581-e77581.
131. Vonghachack, Y., et al. (2015). "Epidemiology of Strongyloides stercoralis on Mekong islands in southern Laos." Acta tropica 141(Pt B): 289-294.
132. Waikagul, J., et al. (2002). "A cross-sectional study of intestinal parasitic infections among schoolchildren in Nan Province, Northern Thailand." The Southeast Asian journal of tropical medicine and public health 33(2): 218-223.
133. Waree, P., et al. (2001). "The present situation of paragonimiasis in endemic area in Phitsanulok Province." The Southeast Asian journal of tropical medicine and public health 32 Suppl 2: 51-54.
134. Warunee, N., et al. (2007). "Intestinal parasitic infections among school children in Thailand." Tropical biomedicine 24(2): 83-88.
135. Whitworth, J. A., et al. (1991). "A field study of the effect of ivermectin on intestinal helminths in man." Transactions of the Royal Society of Tropical Medicine and Hygiene 85(2): 232-234.
136. Widjana, D. P. and P. Sutisna (2000). "Prevalence of soil-transmitted helminth infections in the rural population of Bali, Indonesia." The Southeast Asian journal of tropical medicine and public health 31(3): 454-459.
137. Willcox, H. P. and J. R. Coura (1991). "The efficiency of Lutz, Kato-Katz and Baermann-Moraes (adapted) techniques association to the diagnosis of intestinal helminths." Memorias do Instituto Oswaldo Cruz 86(4): 457-460.
138. Wiria, A.E., et al. (2015). “ [Infection with Soil-Transmitted Helminths Is Associated with Increased Insulin Sensitivity.](https://pubmed.ncbi.nlm.nih.gov/26061042/?from_term=wiria%09plos+one%092015&from_pos=2)”PLoS One. 10(6):e0127746. doi: 10.1371/journal.pone.0127746. eCollection 2015.PMID: 26061042
139. Wondimagegnehu, T., et al. (1992). "Hookworm infection among the Melka Sedi banana plantation residents, middle Awash Valley, Ethiopia." Ethiopian medical journal 30(3): 129-134.
140. Wongjindanon, N., et al. (2005). "Current infection rate of Giardia lamblia in two provinces of Thailand." The Southeast Asian journal of tropical medicine and public health 36 Suppl 4: 21-25.
141. Yaicharoen, R., et al. (2006). "Infection of Blastocystis hominis in primary schoolchildren from Nakhon Pathom province, Thailand." Tropical biomedicine 23(1): 117-122.
142. Yap, P., et al. (2013). "Rapid re-infection with soil-transmitted helminths after triple-dose albendazole treatment of school-aged children in Yunnan, People's Republic of China." The American journal of tropical medicine and hygiene 89(1): 23-31.
143. Yapi, Y. G., et al. (2006). "Prevalence of Geohelminths in Savana and Forest Areas of Côte d'Ivoire." West Afr J Med. 25(2): 124-125.
144. Yelifari, L., et al. (2005). "Distribution of human Oesophagostomum bifurcum, hookworm and Strongyloides stercoralis infections in northern Ghana." Transactions of the Royal Society of Tropical Medicine and Hygiene 99(1): 32-38.
145. Yori, P. P., et al. (2006). "Seroepidemiology of strongyloidiasis in the Peruvian Amazon." The American journal of tropical medicine and hygiene 74(1): 97-102.
146. Zonta, M. L., et al. (2010). "Nutritional status, body composition, and intestinal parasitism among the Mbyá-Guaraní communities of Misiones, Argentina." American journal of human biology : the official journal of the Human Biology Council 22(2): 193-200.
