# Supplementary material for: The Global Prevalence of Strongyloides stercoralis Infection
Source: Pathogens. 2020 Jun 13;9(6):468. doi: 10.3390/pathogens9060468 (PMC7349647; doi:10.3390/pathogens9060468)
Supplement: Supplementary file 1 [file pathogens-09-00468-s001.zip › pathogens-812962-supplementary/pathogens-812962-suppl/Supplementary file S2.docx]

**Review protocol**

To review the existing literature on quantitative data pertaining to strongyloidiasis-related prevalence, incidence, remission/cure, and mortality in humans. Whenever available, the aforementioned data should be age-, sex-, and asymptomatic/symptomatic-specific.

The intention is to explore potential ways to better estimate national and global strogyloidiasis prevalence rates and to deduce also the ivermectin drug demand based on these further advanced estimates.

**Search strategy**

The search strategy is shown in Table 1 with further details on its development and rationale provided below and in Appendix 1. The search strategy basically followed – but further expands and updates – a published systematic review on the global distribution and risk factors for strongyloidiasis [Schär F, Trostdorf U, Giardina F, Khieu V, Muth S, et al. (2013) *Strongyloides stercoralis*: global distribution and risk factors. PLoS Neglected Tropical Diseases 7: e2288.]. No restrictions were set with regard to type of study or publication, location of the work and language of the reference. The bibliographies of all finally included documents were hand-searched for additional references and attempts were made to also access not yet retrieved material such as reports and policy documents.

**Table 1: Summary of the search strategy and retrieved number of raw hits.** The table highlights the databases searched and respective specifications, thesauri, complete search terms with Boolean operators, and the resulting number of raw hits.

| **Database** | **Thesaurus** | **Search terms** | **Boolean** | **Exclusion** | **Number** |
| --- | --- | --- | --- | --- | --- |
|  |  |  | **operators** |  | **of hits** |
|  |  |  |  |  | **(23.05.2017)** |
| PubMed | MeSH | "strongyloides" [MeSH] | OR | published | 2,022 |
|  |  | "strongyloidiasis" [MeSH] |  | before 1990 |  |
| WHOLIS | MeSH | "strongyloides" [MeSH] | OR | published | 0 |
|  |  | "strongyloidiasis" [MeSH] |  | before 1990 |  |
| ISI Web | no thesaurus; | "strongyloides" | OR | published | 6,419 |
| of Science | basic keyword | "strongyloidiasis" |  | before 1990 |  |
| [All Databases] | search |  |  |  |  |

**Organization and screening of literature**

The bibliographic software EndNote (Thomson Reuters Corp., New York City, NY, United States of America) was used to manage all retrieved references. To identify all relevant documents, all duplicates were removed and the remaining references successively screened by title, abstract, and full-text. If in doubt about the relevance of a reference at any step of the screening process, it was included at the respective step and considered at the next and more detailed level (i.e. title screening → abstract screening → full text review).

**Inclusion/Exclusion and extraction of relevant data**

Of particular relevance and hence to be INCLUDED and data to be extracted:

- National estimates (e.g. including national surveys, modelling predictions, but also expert opinion).
- General population/community based surveys (i.e. surveys that are representative for a certain, not overly narrowly defined general population/community (e.g. “communities in the Coastal Region of the country”, “aborigines”, “school children”, “adults”, etc.) and which therefore potentially allow for extrapolations in the respective general population/community).

Not to be considered and therefore to be EXCLUDED and data NOT to be extracted:

- Animal studies (e.g. strongyloidiasis in wild, domestic, or lab animals; NB: the goal is to estimate the global human burden).
- Laboratory studies (NB: the goal is to estimate the global burden in the general human population).
- Health facility based studies (NB: potentially heavily biased and hence difficult to interpret and deduce information on the situation in the general population).
- Case reports/Case series (NB: even if case series of a few tens or hundreds, these data are potentially heavily biased and hence difficult to interpret and deduce information on the situation in the general population).
- Studies in refugees/immigrants/travellers (NB: potentially heavily biased and often also a comparatively small group (see also the just following bullet point!); hence difficult to interpret and deduce information on the situation in the general population; furthermore, here also the question of where the *Strongyloides* infection was acquired poses a particular problem (i.e. location of origin, on the way, location of diagnosis?)).
- Studies in other comparatively narrow defined groups/risk groups (e.g. food handlers, HIV positives, TB patients, sex workers, migrant workers, alcoholics, pregnant women, asthmatics, etc.; NB: such data are only representative for the respective and comparatively narrowly defined group, but the goal is to estimate the global burden in the general human population – however, of note, one could think of including certain large and important groups such as HIV positives particularly in some countries in future work, for instance).

A simple data extraction template will be used (Microsoft Excel version 2010, Microsoft Corp., Redmond, WA, United States of America) to facilitate relevant data extraction (see Appendix 2).

**Expected results and their interpretation**

- Worldwide, the currently available quantitative evidence on the basic epidemiological key parameters (prevalence, incidence, sequelae, cure rate, duration, mortality) of strongyloidiasis.
- Knowledge gaps in these basic epidemiological key parameters.
- Based on all identified evidence, a first more or less sophisticated extrapolation on the total national and global age-, sex, and sequela-specific number of infected individuals.

**Appendix 1: Details on the search strategy development and rationale.**

| **Database** | **Thesaurus** | **Search terms** | **Boolean** | **Number** | **Exclusion** | **Number** |
| --- | --- | --- | --- | --- | --- | --- |
|  |  |  | **operators** | **of hits 1** |  | **of hits 2** |
|  |  |  |  | **(23.05.2017)** |  | **(23.05.2017)** |
| PubMed | no thesaurus; | "strongyloides" | --- | 3,898 | published | 2,799 |
|  | basic keyword |  |  |  | before 1990 |  |
|  | search |  |  |  |  |  |
| PubMed | no thesaurus; | "strongyloidiasis" | --- | 3,745 | published | 2,098 |
|  | basic keyword |  |  |  | before 1990 |  |
|  | search |  |  |  |  |  |
| PubMed | no thesaurus; | "strongyloides" | OR | 5,338 | published | 3,261 |
|  | basic keyword | "strongyloidiasis" |  |  | before 1990 |  |
|  | search |  |  |  |  |  |
| PubMed | MeSH | "strongyloides" | --- | 2,200 | published | 1,565 |
|  |  |  |  |  | before 1990 |  |
| PubMed | MeSH | "strongyloidiasis" | --- | 3,379 | published | 1,785 |
|  |  |  |  |  | before 1990 |  |
| **PubMed** | **MeSH** | **"strongyloides"** | **OR** | **3,864** | **published** | **2,022** |
|  |  | **"strongyloidiasis"** |  |  | **before 1990** |  |
| PubMed | MeSH | "strongyloides" | OR | 2,714 | published | 1,580 |
|  |  | "strongyloidiasis" | AND |  | before 1990 |  |
|  |  | "humans" |  |  |  |  |
| WHOLIS | no thesaurus; | "strongyloides" | --- | 0 | published | 0 |
|  | basic keyword |  |  |  | before 1990 |  |
|  | search |  |  |  |  |  |
| WHOLIS | no thesaurus; | "strongyloidiasis" | --- | 2 | published | 0 |
|  | basic keyword |  |  |  | before 1990 |  |
|  | search |  |  |  |  |  |
| WHOLIS | no thesaurus; | "strongyloides" | OR | 2 | published | 0 |
|  | basic keyword | "strongyloidiasis" |  |  | before 1990 |  |
|  | search |  |  |  |  |  |
| WHOLIS | MeSH | "strongyloides" | --- | 0 | published | 0 |
|  |  |  |  |  | before 1990 |  |
| WHOLIS | MeSH | "strongyloidiasis" | --- | 1 | published | 0 |
|  |  |  |  |  | before 1990 |  |
| **WHOLIS** | **MeSH** | **"strongyloides"** | **OR** | **1** | **published** | **0** |
|  |  | **"strongyloidiasis"** |  |  | **before 1990** |  |
| WHOLIS | MeSH | "strongyloides" | OR | 0 | published | 0 |
|  |  | "strongyloidiasis" | AND |  | before 1990 |  |
|  |  | "humans" |  |  |  |  |
| ISI Web | no thesaurus; | "strongyloides" | --- | 9,134 | published | 6,000 |
| of Science | basic keyword |  |  |  | before 1990 |  |
| [All Databases] | search |  |  |  |  |  |
| ISI Web | no thesaurus; | "strongyloidiasis" | --- | 5,103 | published | 3,228 |
| of Science | basic keyword |  |  |  | before 1990 |  |
| [All Databases] | search |  |  |  |  |  |
| **ISI Web** | **no thesaurus;** | **"strongyloides"** | **OR** | **10,419** | **published** | **6,419** |
| **of Science** | **basic keyword** | **"strongyloidiasis"** |  |  | **before 1990** |  |
| **[All Databases]** | **search** |  |  |  |  |  |

**Appendix 2: Data extraction template.**
