# Supplementary material for: The Global Prevalence of Strongyloides stercoralis Infection
Source: Pathogens. 2020 Jun 13;9(6):468. doi: 10.3390/pathogens9060468 (PMC7349647; doi:10.3390/pathogens9060468)
Supplement: Supplementary file 1 [file pathogens-09-00468-s001.zip › pathogens-812962-supplementary/pathogens-812962-suppl/Supplementary File S3.docx]

**Prevalence adjustment based on diagnostic test**

Formula used to obtain the true prevalence estimate for each study. Formula:

$$\hat{\theta}=\frac{1+sp+\varphi}{se+sp-1}=1-\frac{se-\varphi}{se+sp-1}$$

where $\theta$ is the true prevalence of an imperfect test, $\varphi$ is the expectation of its estimator (i.e. the observed prevalence: the proportion of individuals who test positive), and *se* and *sp* are the test’s sensibility and specificity,
