# Supplementary material for: The Global Prevalence of Strongyloides stercoralis Infection
Source: Pathogens. 2020 Jun 13;9(6):468. doi: 10.3390/pathogens9060468 (PMC7349647; doi:10.3390/pathogens9060468)
Supplement: Supplementary file 1 [file pathogens-09-00468-s001.zip › pathogens-812962-supplementary/pathogens-812962-suppl/Table S1.docx]

Table S1. Strongyloidiasis prevalence (STG-PR) and 95% CI for the countries for which estimates were made

| **Country** | **WHO Region** | **ISO 3** | **STG-PR** |
| --- | --- | --- | --- |
| Afghanistan | EMRO | AFG | 6.7% (3.4% - 10%) |
| Albania | EURO | ALB | 8% (4.1% - 11.9%) |
| Algeria | AFRO | DZA | 4.9% (2.5% - 7.3%) |
| Angola | AFRO | AGO | 9.8% (5% - 14.6%) |
| Antigua and Barbuda | AMRO | ATG | 7.3% (3.7% - 10.8%) |
| Argentina | AMRO | ARG | 5.1% (2.6% - 7.6%) |
| Armenia | EURO | ARM | 6.5% (3.3% - 9.7%) |
| Australia | WPRO | AUS | 0.01% (0.005% - 0.015%) |
| Austria | EURO | AUT | 0.01% (0.005% - 0.015%) |
| Azerbaijan | EURO | AZE | 5.8% (3% - 8.7%) |
| Bahamas | AMRO | BHS | 5.7% (2.9% - 8.5%) |
| Bahrain | EMRO | BHR | 1.9% (1% - 2.9%) |
| Bangladesh | SEARO | BGD | 17.3% (8.8% - 25.7%) |
| Barbados | AMRO | BRB | 8.6% (4.4% - 12.8%) |
| Belarus | EURO | BLR | 6.5% (3.3% - 9.7%) |
| Belgium | EURO | BEL | 0.02% (0.01% - 0.03%) |
| Belize | AMRO | BLZ | 11.8% (6% - 17.6%) |
| Benin | AFRO | BEN | 10.7% (5.5% - 16%) |
| Bhutan | SEARO | BTN | 18.5% (9.4% - 27.6%) |
| Bolivia (Plurinational State of) | AMRO | BOL | 10.4% (5.3% - 15.4%) |
| Bosnia and Herzegovina | EURO | BIH | 9.4% (4.8% - 14.1%) |
| Botswana | AFRO | BWA | 6.3% (3.2% - 9.3%) |
| Brazil | AMRO | BRA | 11.2% (5.7% - 16.6%) |
| Brunei Darussalam | WPRO | BRN | 11.9% (6.1% - 17.7%) |
| Bulgaria | EURO | BGR | 6.3% (3.2% - 9.3%) |
| Burkina Faso | AFRO | BFA | 9.5% (4.8% - 14.1%) |
| Burundi | AFRO | BDI | 11.7% (6% - 17.4%) |
| Cabo Verde | AFRO | CPV | 5.8% (3% - 8.7%) |
| Cambodia | WPRO | KHM | 13.6% (6.9% - 20.3%) |
| Cameroon | AFRO | CMR | 12.8% (6.5% - 19%) |
| Canada | AMRO | CAN | 0% (0% - 0%) |
| Central African Republic | AFRO | CAF | 11.9% (6.1% - 17.7%) |
| Chad | AFRO | TCD | 8.2% (4.2% - 12.2%) |
| Chile | AMRO | CHL | 11.2% (5.7% - 16.7%) |
| China | WPRO | CHN | 6.6% (3.3% - 9.8%) |
| Colombia | AMRO | COL | 18.4% (9.4% - 27.4%) |
| Comoros | AFRO | COM | 9.9% (5% - 14.7%) |
| Congo | AFRO | COG | 13.4% (6.9% - 20%) |
| Costa Rica | AMRO | CRI | 15.7% (8% - 23.4%) |
| Croatia | EURO | HRV | 8.6% (4.4% - 12.8%) |
| Côte d'Ivoire | AFRO | CIV | 11.6% (5.9% - 17.3%) |
| Cuba | AMRO | CUB | 9.5% (4.9% - 14.2%) |
| Cyprus | EURO | CYP | 2.8% (1.4% - 4.2%) |
| Czech Republic | EURO | CZE | 4.5% (2.3% - 6.7%) |
| Democratic People's Republic of Korea | SEARO | PRK | 9.5% (4.8% - 14.2%) |
| Democratic Republic of the Congo | AFRO | COD | 13% (6.6% - 19.4%) |
| Denmark | EURO | DNK | 0% (0% - 0%) |
| Djibouti | EMRO | DJI | 6.8% (3.5% - 10.1%) |
| Dominica | AMRO | DMA | 14.2% (7.2% - 21.1%) |
| Dominican Republic | AMRO | DOM | 10.3% (5.3% - 15.4%) |
| Ecuador | AMRO | ECU | 14.5% (7.4% - 21.6%) |
| Egypt | EMRO | EGY | 4.9% (2.5% - 7.3%) |
| El Salvador | AMRO | SLV | 12.1% (6.2% - 18.1%) |
| Equatorial Guinea | AFRO | GNQ | 13.5% (6.9% - 20.1%) |
| Eritrea | AFRO | ERI | 8.3% (4.2% - 12.3%) |
| Estonia | EURO | EST | 4.1% (2.1% - 6.2%) |
| Ethiopia | AFRO | ETH | 10.2% (5.2% - 15.2%) |
| Fiji | WPRO | FJI | 15.9% (8.1% - 23.7%) |
| Finland | EURO | FIN | 0% (0% - 0%) |
| France | EURO | FRA | 0.02% (0.01% - 0.03%) |
| French Guiana | AMRO | GUF | 13.2% (6.7% - 19.7%) |
| Gabon | AFRO | GAB | 12.9% (6.6% - 19.2%) |
| Gambia | AFRO | GMB | 9.5% (4.8% - 14.1%) |
| Georgia | EURO | GEO | 10% (5.1% - 14.8%) |
| Germany | EURO | DEU | 0.1% (0% - 0.1%) |
| Ghana | AFRO | GHA | 11.1% (5.7% - 16.6%) |
| Greece | EURO | GRC | 0.4% (0.2% - 0.6%) |
| Grenada | AMRO | GRD | 13.7% (7% - 20.4%) |
| Guatemala | AMRO | GTM | 13.7% (7% - 20.4%) |
| Guinea | AFRO | GIN | 13.1% (6.7% - 19.5%) |
| Guinea-Bissau | AFRO | GNB | 12.7% (6.5% - 18.9%) |
| Guyana | AMRO | GUY | 14.6% (7.5% - 21.8%) |
| Haiti | AMRO | HTI | 12.3% (6.3% - 18.4%) |
| Honduras | AMRO | HND | 13.8% (7.1% - 20.6%) |
| Hungary | EURO | HUN | 4.9% (2.5% - 7.3%) |
| Iceland | EURO | ISL | 0% (0% - 0%) |
| India | SEARO | IND | 10.4% (5.3% - 15.4%) |
| Indonesia | SEARO | IDN | 16.4% (8.3% - 24.4%) |
| Iran (Islamic Republic of) | EMRO | IRN | 4.8% (2.5% - 7.2%) |
| Iraq | EMRO | IRQ | 5.3% (2.7% - 7.9%) |
| Ireland | EURO | IRL | 0% (0% - 0%) |
| Israel | EURO | ISR | 0.1% (0% - 0.1%) |
| Italy | EURO | ITA | 0.03% (0.01% - 0.04%) |
| Jamaica | AMRO | JAM | 13.4% (6.8% - 20%) |
| Japan | WPRO | JPN | 0.04% (0.02% - 0.06%) |
| Jordan | EMRO | JOR | 4.4% (2.3% - 6.6%) |
| Kazakhstan | EURO | KAZ | 3.3% (1.7% - 4.9%) |
| Kenya | AFRO | KEN | 8.7% (4.5% - 13%) |
| Kiribati | WPRO | KIR | 6% (3.1% - 8.9%) |
| Kuwait | EMRO | KWT | 1% (0.5% - 1.5%) |
| Kyrgyzstan | EURO | KGZ | 6.5% (3.3% - 9.6%) |
| Lao People's Democratic Republic | WPRO | LAO | 13.4% (6.8% - 20%) |
| Latvia | EURO | LVA | 5.1% (2.6% - 7.5%) |
| Lebanon | EMRO | LBN | 6.4% (3.3% - 9.5%) |
| Lesotho | AFRO | LSO | 9.3% (4.7% - 13.8%) |
| Liberia | AFRO | LBR | 16.5% (8.4% - 24.6%) |
| Libya | EMRO | LBY | 4.2% (2.1% - 6.3%) |
| Lithuania | EURO | LTU | 5% (2.5% - 7.4%) |
| Luxembourg | EURO | LUX | 0% (0% - 0%) |
| Madagascar | AFRO | MDG | 13.4% (6.8% - 20%) |
| Malawi | AFRO | MWI | 11.2% (5.7% - 16.8%) |
| Malaysia | WPRO | MYS | 15.9% (8.1% - 23.7%) |
| Maldives | SERAO | MDV | 0.1% (0.1% - 0.2%) |
| Mali | AFRO | MLI | 7.7% (3.9% - 11.5%) |
| Malta | EURO | MLT | 0.02% (0.01% - 0.03%) |
| Mauritania | AFRO | MRT | 6.7% (3.4% - 10%) |
| Mauritius | AFRO | MUS | 13.1% (6.7% - 19.5%) |
| Mexico | AMRO | MEX | 7% (3.6% - 10.5%) |
| Micronesia (Federated States of) | WPRO | FSM | 8% (4.1% - 11.9%) |
| Mongolia | WPRO | MNG | 4.3% (2.2% - 6.4%) |
| Morocco | EMRO | MAR | 5.9% (3% - 8.7%) |
| Mozambique | AFRO | MOZ | 10.8% (5.5% - 16.1%) |
| Myanmar | SEARO | MMR | 19.2% (9.8% - 28.6%) |
| Namibia | AFRO | NAM | 6.4% (3.2% - 9.5%) |
| Nepal | SEARO | NPL | 14.2% (7.2% - 21.1%) |
| Netherlands | EURO | NLD | 0.1% (0% - 0.1%) |
| New Caledonia | WPRO | NCL | 0% (0% - 0%) |
| New Zealand | WPRO | NZL | 0.04% (0.02% - 0.06%) |
| Nicaragua | AMRO | NIC | 15% (7.6% - 22.3%) |
| Niger | AFRO | NER | 7.6% (3.9% - 11.4%) |
| Nigeria | AFRO | NGA | 10.7% (5.4% - 15.9%) |
| Norway | EURO | NOR | 0% (0% - 0%) |
| Oman | EMRO | OMN | 3% (1.6% - 4.5%) |
| Pakistan | EMRO | PAK | 7.5% (3.8% - 11.2%) |
| Panama | AMRO | PAN | 15.7% (8% - 23.4%) |
| Papua New Guinea | WPRO | PNG | 19.4% (9.9% - 28.9%) |
| Paraguay | AMRO | PRY | 9.1% (4.6% - 13.6%) |
| Peru | AMRO | PER | 12.5% (6.4% - 18.6%) |
| Philippines | WPRO | PHL | 15% (7.7% - 22.4%) |
| Poland | EURO | POL | 5% (2.5% - 7.4%) |
| Portugal | EURO | PRT | 0.05% (0.06% - 0.07%) |
| Puerto Rico | AMRO | PRI | 9.1% (4.6% - 13.5%) |
| Qatar | EMRO | QAT | 0% (0% - 0%) |
| Republic of Korea | WPRO | KOR | 6.8% (3.5% - 10.2%) |
| Republic of Moldova | EURO | MDA | 6.2% (3.2% - 9.3%) |
| Romania | EURO | ROU | 6.1% (3.1% - 9.1%) |
| Russian Federation | EURO | RUS | 4.1% (2.1% - 6.1%) |
| Rwanda | AFRO | RWA | 11.2% (5.7% - 16.8%) |
| Saint Lucia | AMRO | LCA | 13.8% (7% - 20.5%) |
| Saint Vincent and the Grenadines | AMRO | VCT | 10.8% (5.5% - 16.2%) |
| Samoa | WPRO | WSM | 5% (2.5% - 7.5%) |
| Sao Tome and Principe | AFRO | STP | 20.7% (10.6% - 30.8%) |
| Saudi Arabia | EMRO | SAU | 2% (1% - 3%) |
| Senegal | AFRO | SEN | 8.7% (4.4% - 13%) |
| Seychelles | AFRO | SYC | 12.5% (6.4% - 18.7%) |
| Sierra Leone | AFRO | SLE | 17% (8.7% - 25.3%) |
| Singapore | WPRO | SGP | 6.2% (3.2% - 9.3%) |
| Slovakia | EURO | SVK | 6% (3.1% - 8.9%) |
| Slovenia | EURO | SVN | 7.3% (3.7% - 10.9%) |
| Solomon Islands | WPRO | SLB | 19% (9.7% - 28.4%) |
| Somalia | EMRO | SOM | 7.9% (4% - 11.7%) |
| South Africa | AFRO | ZAF | 6.4% (3.3% - 9.6%) |
| South Sudan | AFRO | SSD | 11% (5.6% - 16.4%) |
| Spain | EURO | ESP | 0.2% (0.1% - 0.3%) |
| Sri Lanka | SEARO | LKA | 11.7% (6% - 17.4%) |
| Sudan | EMRO | SDN | 7.1% (3.6% - 10.6%) |
| Suriname | AMRO | SUR | 14.5% (7.4% - 21.6%) |
| Swaziland | AFRO | SWZ | 8.6% (4.4% - 12.9%) |
| Sweden | EURO | SWE | 0% (0% - 0%) |
| Switzerland | EURO | CHE | 0.01% (0.005% - 0.015%) |
| Syrian Arab Republic | EMRO | SYR | 5.7% (2.9% - 8.5%) |
| Tajikistan | EURO | TJK | 7.8% (4% - 11.6%) |
| Thailand | SEARO | THA | 10.8% (5.5% - 16.1%) |
| The former Yugoslav Republic of Macedonia | EURO | MKD | 6.7% (3.4% - 10%) |
| Togo | AFRO | TGO | 11.3% (5.8% - 16.8%) |
| Tonga | WPRO | TON | 5% (2.5% - 7.5%) |
| Trinidad and Tobago | AMRO | TTO | 12% (6.1% - 17.9%) |
| Tunisia | EMRO | TUN | 5% (2.6% - 7.5%) |
| Turkey | EURO | TUR | 5.6% (2.9% - 8.4%) |
| Turkmenistan | EURO | TKM | 3.9% (2% - 5.8%) |
| Uganda | AFRO | UGA | 11.5% (5.9% - 17.1%) |
| Ukraine | EURO | UKR | 6.6% (3.4% - 9.9%) |
| United Arab Emirates | EMRO | ARE | 0% (0% - 0%) |
| United Kingdom of Great Britain and Northern Ireland | EURO | GBR | 0% (0% - 0%) |
| United Republic of Tanzania | AFRO | TZA | 10.9% (5.6% - 16.3%) |
| United States of America | AMRO | USA | 0.01% (0.005% - 0.015%) |
| United States Virgin Islands | AMRO | VIR | 0% (0% - 0%) |
| Uruguay | AMRO | URY | 8.7% (4.4% - 12.9%) |
| Uzbekistan | EURO | UZB | 4.7% (2.4% - 7%) |
| Vanuatu | WPRO | VUT | 0.3% (0.2% - 0.4%) |
| Venezuela (Bolivarian Republic of) | AMRO | VEN | 11.2% (5.7% - 16.7%) |
| Viet Nam | WPRO | VNM | 13.1% (6.7% - 19.6%) |
| Western Sahara | AFRO | ESH | 5% (2.5% - 7.5%) |
| Yemen | EMRO | YEM | 6.2% (3.2% - 9.2%) |
| Zambia | AFRO | ZMB | 10.5% (5.3% - 15.6%) |
| Zimbabwe | AFRO | ZWE | 8.7% (4.4% - 13%) |

ISO 3: International Organization for Standardization country code
