# Supplementary material for: The Global Prevalence of Strongyloides stercoralis Infection
Source: Pathogens. 2020 Jun 13;9(6):468. doi: 10.3390/pathogens9060468 (PMC7349647; doi:10.3390/pathogens9060468)
Supplement: Supplementary file 1 [file pathogens-09-00468-s001.zip › pathogens-812962-supplementary/pathogens-812962-suppl/Table S2.docx]

**Table S1**. Summary of tested STAR models to assess the relationship of STG-PR with model variables.

| GDP | GDPHealth | EDU | RURAL | CROP | FOREST | RUG | SANIT | TEMP | RAIN | REGION_RND_ | AIC | Rank |
| --- | --- | --- | --- | --- | --- | --- | --- | --- | --- | --- | --- | --- |
| X | - | - | X | - | - | X | X | X | X | X | 1322.1 | 1 |
| X | - | X | X | - | - | X | X | X | X | X | 1343.2 | 2 |
| X |  | X | X | X | - | X | X | X | X | X | 1378.6 | 3 |
| - | X | X | X | X | X | - | X | X | X | X | 1420.7 | 4 |
| - | X | - | X | X | X | - | X | X | X | X | 1455.7 | 5 |

X: variable included in the model; -: variable excluded from the model

The table shows the first 5 models ranked by their AIC. The first model (lowest AIC) was used to estimate STG-PR per each country. GDP is per capita gross domestic product, GDPHealth is the percentage of GDP allocated to health expenditure, EDU is the percentage of the population who attended primary education, RURAL is the percentage of the population living in a rural setting, CROP is the percentage of the country’s land allocated for agriculture, FOREST is the percentage of the country’s land covered by forest, RUG is the ruggedness index of the country, SANIT is the percentage of the population with access to a proper latrine, TEMP is the mean annual temperature, RAIN is the total annual rainfall, and REGION_RND_ is the region as a random effect.
